# Low-dose ionizing radiation *in vivo* unlocks the therapeutic potential of prevascularized dermal spheroids in chronic wounds

**DOI:** 10.1016/j.mtbio.2025.102561

**Published:** 2025-11-15

**Authors:** Filipe Rocha, Inês Sofia Vala, Paula de Oliveira, Pedro Faísca, Carolina Fernandes, Marta Teixeira Pinto, Filomena Pina, Esmeralda Poli, Isabel Diegues, Eugénia Carvalho, Cristina C. Barrias, Susana Constantino Rosa Santos

**Affiliations:** a Centro Cardiovascular da Universidade de Lisboa (CCUL@RISE), Faculdade de Medicina, Universidade de Lisboa, Portugal; b Gulbenkian Institute for Molecular Medicine, Oeiras, Portugal; c i3s - Institute for Research and Innovation in Health, University of Porto, Portugal; d Hospital Santa Maria, Unidade Local de Saúde Santa Maria (ULSSM), Portugal; e CNC-UC - Center for Neuroscience and Cell Biology, University of Coimbra. CIBB - Center for Inovative Biomedicine and Biotechnology, Institute for Interdisciplinary Research, University of Coimbra, Portugal; f ICBAS-Instituto de Ciências Biomédicas de Abel Salazar, Universidade do Porto, Portugal; g INEB-Instituto de Engenharia Biomédica, Universidade do Porto, Portugal

**Keywords:** Wound healing, Fibroblasts, Endothelial cells, Spheroids, Low-dose ionizing radiation, Chronic wounds, Angiogenesis

## Abstract

Chronic wounds (CWs), particularly in diabetic patients, remain a major clinical challenge because of impaired angiogenesis and fibroblast dysfunction. This study investigated the therapeutic synergy between low-dose ionizing radiation (LDIR; 0.3 Gy) and prevascularized spheroids composed of human dermal fibroblasts (HDFs) and endothelial colony-forming cells (ECFCs) embedded in a xeno-free fibrin hydrogel. *In vitro*, LDIR enhanced endothelial sprouting and fibroblast outgrowth from HDF-ECFC spheroids without affecting morphology or viability. Gene and protein analyses revealed LDIR-induced upregulation of angiogenic and profibrotic markers, including VEGFR2, CD31, VE-cadherin, COL4A2, α-SMA, TGF-β1 and LH2. Conditioned media from irradiated spheroids exhibited a proangiogenic secretome and significantly increased neovascularization in the chorioallantoic membrane assay. Notably, spheroids composed exclusively of fibroblasts failed to exhibit these LDIR-induced effects, highlighting the importance of endothelial–stromal interactions. Importantly, *in vivo*, the combination of nonirradiated HDF-ECFC spheroids followed by local LDIR significantly accelerated wound healing and restored skin remodeling, skin architecture and histological integrity by reducing pathological skin remodeling in the *db/db* diabetic mouse model. In contrast, neither LDIR *per se* nor preirradiated spheroids on their own promoted wound closure or histological repair. These findings demonstrate that LDIR enhances the regenerative capacity of prevascularized dermal spheroids through molecular activation, paracrine signaling and stromal‒endothelial crosstalk but requires *in vivo* irradiation of the wound niche to achieve therapeutic benefit. This combinatorial strategy offers a clinically translatable approach to modulate the wound microenvironment and overcome current limitations in CW management.

## Highlights

• LDIR enhances endothelial sprouting and fibroblast outgrowth from HDF-ECFC spheroids.
• LDIR activates angiogenic and profibrotic programs at the transcript and protein levels.
• LDIR potentiates the angiogenic secretome and promotes *in vivo* neovascularization.
• *In vivo* irradiation is required to unlock the therapeutic effect of implanted spheroids.
• Fibrin-embedded HDF-ECFC spheroids with LDIR improve healing in diabetic wounds.

## Graphical abstract

**Figure:**
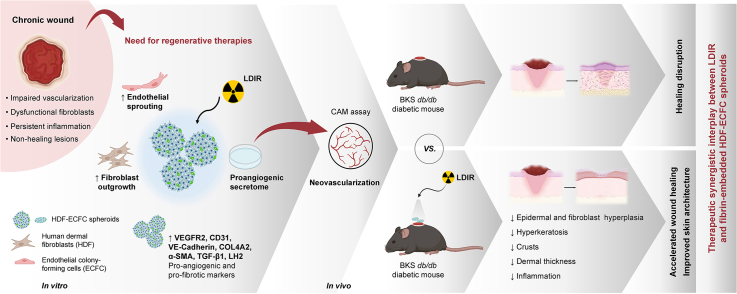
Schematic flowchart of the synergistic action of LDIR and prevascularized HDF-ECFC spheroids in promoting chronic wound healing in diabetic models.

## 1 Introduction

Chronic wounds (CWs), particularly diabetic foot ulcers, represent a major clinical and socioeconomic burden, impacting approximately a quarter of individuals with diabetes [1]. These wounds are characterized by persistent inflammation, impaired angiogenesis, dysfunctional fibroblast activity and disorganized extracellular matrix (ECM) remodeling [[2], [3], [4]]. These pathological features disrupt normal healing progression with a heightened risk of infections, lower-extremity amputations, including minor and major procedures, and increased mortality rates within the first year of diagnosis [1,2,5,6]. Although current wound care strategies have improved symptom control and infection management, regenerative therapies that can simultaneously target vascular and structural tissue remodeling are urgently needed [2,7].

Physiological wound healing is a tightly regulated process comprising overlapping phases of hemostasis, inflammation, proliferation, and remodeling [2,3,8,9]. Central to the proliferative phase are endothelial cells and fibroblasts, which orchestrate neovascularization and ECM deposition, respectively. Upon activation, fibroblasts can differentiate into contractile myofibroblasts, which secrete structural ECM proteins and mediate wound contraction through α-smooth muscle actin (α-SMA) expression [2,3,[8], [9], [10]]. However, in CWs, excessive oxidative stress, proteolytic imbalance and impaired intracellular signaling disrupt this coordination, leading to nonhealing lesions [2,3,9].

To address these challenges, three-dimensional (3D) coculture systems incorporating human dermal fibroblasts (HDFs) and endothelial colony-forming cells (ECFCs) have emerged as promising platforms for recapitulating the cell‒cell and cell‒matrix interactions of native tissue [[11], [12], [13], [14]]. When embedded in fibrin hydrogels, these HDF-ECFC spheroids exhibit morphogenetic activity, including endothelial sprouting and matrix remodeling, suggesting the potential for therapeutic neovascularization [12]. Importantly, the applicability of human cell-based constructs in murine models has been demonstrated, as both autologous mouse skin constructs and allogeneic human skin constructs have been shown to facilitate wound closure and promote full-thickness skin regeneration when applied to excisional wounds [15]. These findings support the immunotolerability and functional relevance of using human spheroid-based biomaterials in preclinical mouse models. Nonetheless, their responsiveness to pathological microenvironments and their functional efficacy *in vivo* remains insufficiently explored, particularly in the context of CW.

Modular tissue engineering has also emerged as a promising approach to generate dense tissues through the co-assembly of microtissue units (spheroids) as building blocks [[16], [17], [18]]. These can be obtained by directed cell aggregation in non-adhesive microwell arrays, which allow rapid and reproducible production of large quantities of uniform, scaffold-free spheroids, a practical advantage for clinical translation [12]. Compared with complex or multilayered cell sheets, spheroids offer a significantly higher surface-area-to-volume ratio, reducing diffusional limitations and improving nutrient and oxygen exchange [12,19]. Moreover, spheroids act as endogenous growth-factor reservoirs, releasing a broad range of pro-regenerative cytokines at physiologically relevant levels [16]. This multimodal paracrine activity enables the coordinated activation of angiogenic, fibroproliferative, and remodeling pathways, whereas topical delivery of single cytokines or recombinant growth factors has shown limited efficacy due to rapid degradation, restricted diffusion, and the inability to sustain multi-pathway activation [3,16,20].

Low-dose ionizing radiation (LDIR; ≤0.8 Gy) has gained attention as a biological modulator of tissue repair [[21], [22], [23], [24], [25], [26]]. Unlike moderate and high-dose exposure (>1 Gy), LDIR is noncytotoxic and instead exerts positive effects, including activation of endothelial cells, promotion of angiogenesis and attenuation of inflammation [[21], [22], [23], [24], [25], [26], [27], [28]]. Preclinical models have demonstrated its ability to increase vascular density [[21], [22], [23], [24], [25], [26]] and modulate immune responses in ischemic tissues [24]. These properties position LDIR as a promising, clinically accessible adjunct in regenerative medicine. However, its integration with advanced bioengineered constructs remains largely unexplored.

In this study, we investigated a novel synergistic strategy that leverages the biological modulatory effects of LDIR to enhance the therapeutic efficacy of prevascularized dermal spheroids. Using a clinically relevant 0.3 Gy electron beam dose, we assessed the cellular, molecular and paracrine responses of spheroids *in vitro* and evaluated their regenerative performance in the *db/db* diabetic mouse model of CW. This approach aims to potentiate both the angiogenic and reparative capacity of spheroid-based constructs through LDIR, offering a synergistic platform to address the multifaceted pathophysiology of CW.

## 2 Results

### 2.1 LDIR enhances endothelial sprouting and promotes fibroblast outgrowth from HDF-ECFC spheroids

**Fig. 1:**
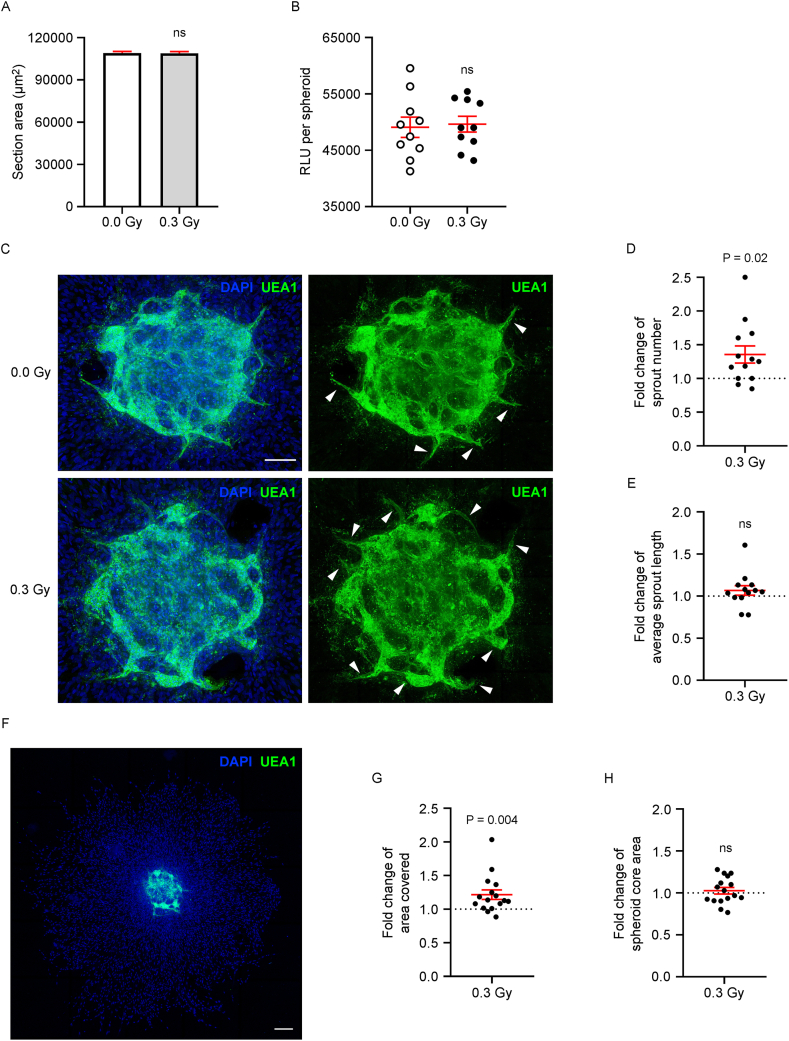
LDIR enhances endothelial sprouting and promotes fibroblast outgrowth from HDF-ECFC spheroids. HDFs and ECFCs were coseeded at a 5:1 ratio in agarose microwell molds to form HDF-ECFC spheroids within 24 h (day 0). The spheroids were then exposed to LDIR (0.3 Gy) or sham irradiation (0.0 Gy). On day 1 of maturation: (**A**) Spheroid size was measured as the section area from top-view images (μm^2^; n = 29 spheroids per group). (**B**) Spheroid metabolic activity was assessed via a luminescence-based ATP quantification assay. Luminescence (RLU) was normalized to the number of spheroids per replicate (n = 10 sets of 8–10 spheroids/group). (**C-H**) Spheroids were embedded in fibrin (Fb) hydrogel for 72 h and analyzed by immunofluorescence. (**C**) Representative images showing ECFC sprouting (UEA-1, green) under 0.0 Gy and 0.3 Gy conditions. The DNA was counterstained with DAPI (blue). The white arrows indicate sprouts extending beyond the spheroid boundary. Scale bar: 100 μm. (**D**) Quantification of the sprout number in irradiated spheroids normalized to that in sham controls (n = 13 spheroids/group). (**E**) Average sprout length expressed as the fold change relative to that in sham-irradiated controls (n = 13). (**F**) Representative image of HDF migration from spheroids after 72 h in the Fb hydrogel. ECFCs were stained with UEA-1 (green), and DNA with DAPI (blue). Cells positive only for DAPI were considered HDFs. Scale bar: 400 μm. (**G**) Quantification of the migration area per irradiated spheroid normalized to that of sham controls (n = 15). (**H**) Spheroid core area expressed as the fold change in irradiated vs. sham spheroids (n = 15). (**A**, **B, D, E, G, H**) Individual data and/or the mean ± SEM (in red) are shown. Parametric statistics were applied assuming a normal distribution: (**A, B**) unpaired, (**D, E, G, H**) paired two-tailed t-tests. Significant P values are shown; ns = not significant. (For interpretation of the references to colour in this figure legend, the reader is referred to the Web version of this article.)

To evaluate the effects of LDIR on prevascularized spheroids, HDFs and ECFCs were co-seeded at a 5:1 ratio in agarose microwell molds [12]. Compact spheroids formed within 24 h and were exposed to a single 0.3 Gy (day 0) dose via a 9 MeV electron beam, a clinically relevant modality for superficial tissue irradiation. The control spheroids underwent sham irradiation. On day 1 postirradiation, spheroid size and ATP content were assessed between the groups (Fig. 1A and B), and the results revealed no significant differences in morphology or metabolic activity.

To assess endothelial sprouting, spheroids were embedded in fibrin hydrogels on day 1 postirradiation and cultured for 72 h. Confocal microscopy revealed a significant increase in ECFC sprout number in irradiated spheroids (9.62 ± 2.43 vs. 7.62 ± 2.63; illustrated in Fig. 1C and quantified in Fig. 1D), with no difference in sprout length (105.16 ± 15.15 μm vs. 100.11 ± 14.46 μm; Fig. 1E). Fibroblast extension into the surrounding matrix was also increased in the irradiated group, with a larger radial outgrowth area (5.14 ± 0.92 mm^2^) than in the sham control group (4.33 ± 0.82 mm^2^), as illustrated in Fig. 1F and quantified in Fig. 1G. The size of the spheroid core remained unchanged (Fig. 1H), suggesting that LDIR does not induce significant changes in the morphology of the spheroid core.

These results demonstrate that LDIR potentiates both endothelial sprouting and fibroblast outgrowth from HDF-ECFC spheroids.

### 2.2 LDIR triggers angiogenic and profibrotic signatures in HDF-ECFC spheroids

**Fig. 2:**
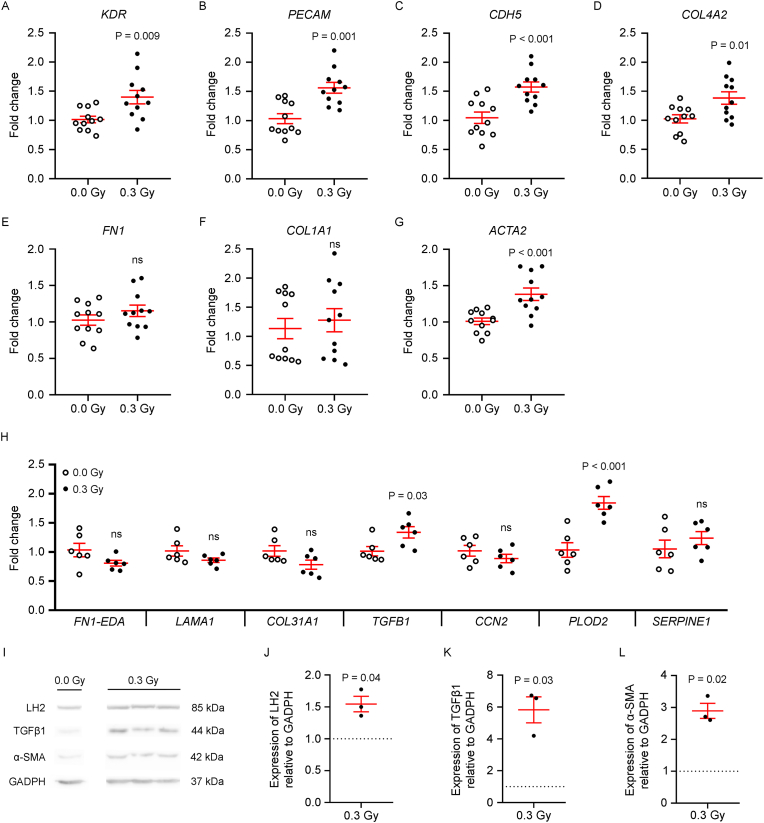
LDIR triggers angiogenic and profibrotic gene and protein signatures in HDF-ECFC spheroids. HDFs and ECFCs were coseeded at a 5:1 ratio in agarose microwell molds to form HDF-ECFC spheroids within 24 h (day 0). The spheroids were then exposed to LDIR (0.3 Gy) or sham irradiation (0.0 Gy). On day 1 of maturation (d1), the gene expression levels of (**A**) *KDR*, (**B**) *PECAM1*, (**C**) *CDH5*, (**D**) *COL4A2*, (**E**) *FN1*, (**F**) *COL1A1*, (**G**) *ACTA2* (n = 11 spheroids/group), and (**H**) *FN1-EDA*, *LAMA1*, *COL3A1*, *TGFB1*, *CCN2*, *PLOD2*, and *SERPINE1* (n = 6/group) were quantified via qRT‒PCR. (**A–H**) Relative expression values were obtained by normalization to 18S and are presented as the fold change over the mean expression in sham-irradiated controls. (**I-L**) At the protein level, spheroids (81 per condition) were lysed and analyzed by Western blotting. (**I**) Representative blots showing the expression of LH2, TGF-β1, and α-SMA, with GAPDH used as a loading control. The 0.0 Gy and 0.3 Gy conditions were analyzed in 1 and 3 sets of 81 spheroids, respectively. (**J–L**) The protein expression of LH2, TGF-β1, and α-SMA was normalized to that of GAPDH and is presented as the fold change over the sham-irradiated controls. (**A-H, J-L**) Individual data and the mean ± SEM (in red) are shown. For statistical comparisons, unpaired two-tailed t tests were used when the data were normally distributed (**A, C–E, G, H**), Mann–Whitney tests were used when they were not (**B, F**), and one-sample t-tests were used for comparisons with a hypothetical mean of 1 (**J–L**). Significant P values are displayed; ns = not significant. (For interpretation of the references to colour in this figure legend, the reader is referred to the Web version of this article.)

To investigate the molecular basis of LDIR-induced sprouting and fibroblast migration, gene expression in HDF-ECFC spheroids was analyzed via qRT‒PCR. LDIR significantly upregulated proangiogenic genes, including *KDR* (VEGFR2), *PECAM1* (CD31), *CDH5* (VE-cadherin), and *COL4A2* (encoding a vascular basement membrane component; Fig. 2A–D). The expression of *FN1* (fibronectin) and *COL1A1* (collagen type I) remained unchanged (Fig. 2E and F), whereas that of *ACTA2* (encoding α-smooth muscle actin, α-SMA) was markedly increased, suggesting early features of a myofibroblast-like phenotype (Fig. 2G). Additional myofibroblast-associated genes, including *TGFB1* (transforming growth factor-β1) and *PLOD2* (procollagen-lysine 2-oxoglutarate 5-dioxygenase 2), were also induced, whereas *FN1*-*EDA* (fibronectin 1 extra domain A isoform), *LAMA1* (laminin subunit alpha-1), *COL3A1* (collagen type III), *CCN2* (cellular communication network factor 2), and *SERPINE1* (plasminogen activator inhibitor 1) showed no significant changes (Fig. 2H).

Protein expression was evaluated by Western blot analysis of pooled spheroid lysates (n = 81 per group) collected 24 h postirradiation. Compared with those of GAPDH, increased levels of LH2 (encoded by *PLOD2*), TGF-β1, and α-SMA (encoded by *ACTA2*) were detected, confirming the activation of profibrotic pathways at the protein level (Fig. 2I–L). These data demonstrate that LDIR promotes a molecular response in HDF-ECFC spheroids that encompasses both angiogenic and profibrotic features.

### 2.3 LDIR induces partial myofibroblastic transcriptional priming in HDF monoculture spheroids

**Fig. 3:**
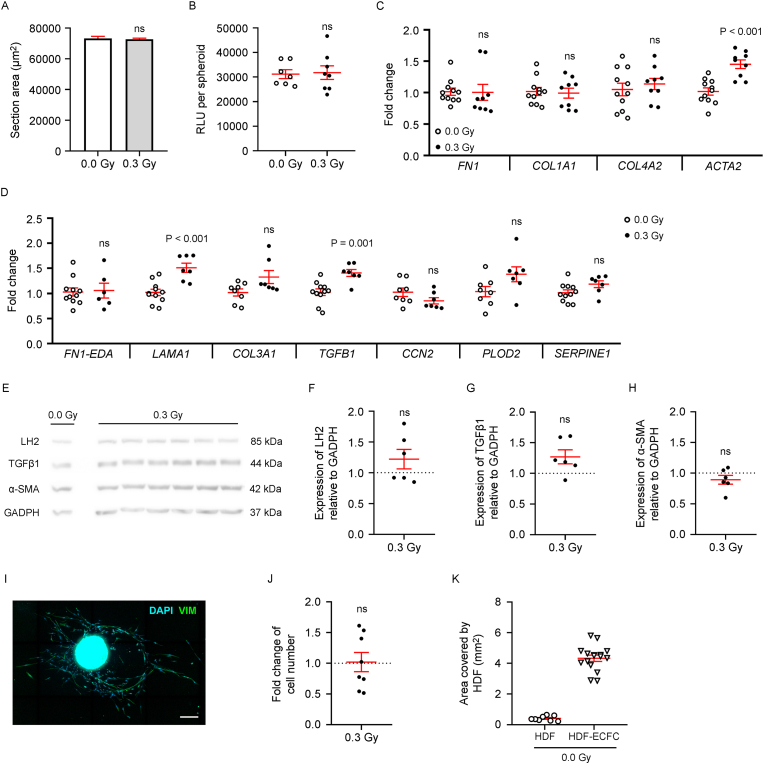
LDIR induces partial myofibroblastic transcriptional priming in HDF monoculture spheroids. HDF spheroids formed in agarose microwell molds within 24 h (day 0) and were exposed to LDIR (0.3 Gy) or sham irradiation (0.0 Gy). On day 1 (d1) of maturation, (**A**) spheroid size was quantified as the section area from the top-view images (μm^2^; n = 26 for 0.0 Gy and n = 57 for 0.3 Gy). (**B**) Spheroid metabolism was assessed via ATP content via a luminescence assay in the sham (n = 7 sets of 8–10 spheroids) and LDIR (n = 8 sets of 8–10 spheroids) groups. (**C**–**D**) Gene expression levels of (**C**) *FN1, COL1A1, COL4A2* and *ACTA2* (n = 11 for 0.0 Gy, n = 9 for 0.3 Gy) and (**D**) *FN1-EDA, LAMA1*, *COL3A1*, *TGFβ1*, *CCN2*, *PLOD2* and *SERPINE1* (n = 8–11 for 0.0 Gy, n = 6 for 0.3 Gy) were evaluated via qRT‒PCR. Cycle threshold values were normalized to 18S values, and the data are presented as the fold change relative to the sham group. (**E**–**H**) At d1, spheroids were lysed and analyzed by Western blot for protein expression of LH2, TGF-β1, α-SMA, and GAPDH (E) under sham and LDIR conditions (1 and 6 sets of 81 spheroids, respectively). (**F–H**) Protein levels were normalized to those of GAPDH and are expressed as the fold change relative to those in the sham group. (**I**–**K**) At d1, spheroids were embedded in fibrin (Fb) hydrogels for 72 h and analyzed by immunofluorescence. (**I**) Representative image of HDF migration in Fb hydrogels (vimentin: green, DAPI: blue). Scale bar: 200 μm. (**J**) The number of extraspheroid cells was quantified and normalized to that in the sham group (n = 8 per group). (**K**) The area occupied by extraspheroid HDFs was quantified in HDF spheroids (n = 8) and HDF-ECFC spheroids (n = 16) under control conditions (0.0 Gy). (**A**-**D**, **F**-**H**, **J**, **K**) Individual data and/or the mean ± SEM (red) are shown. Statistical comparisons were performed via (**A**-**D**, **K**) unpaired two-tailed t-tests or Mann–Whitney tests depending on data normality or (**J**) paired two-tailed t-tests and (**F-H**) one-sample t-tests with a hypothetical mean = 1. (**A**-**D**, **F**-**H**, **J**, **K**) Significant P values are displayed; ns = not significant. (For interpretation of the references to colour in this figure legend, the reader is referred to the Web version of this article.)

To evaluate the direct effects of LDIR on fibroblast behavior in the absence of endothelial influence, monoculture spheroids composed exclusively of HDFs were generated in agarose microwell molds. After 24 h of aggregation, compact spheroids were exposed or not to 0.3 Gy (day 0). Twenty-four hours later (day 1), neither spheroid size nor ATP content was significantly altered by LDIR (Fig. 3A and B). Gene expression analysis revealed significant upregulation of *ACTA2*, *LAMA1* and *TGFB1*, with trends toward increased *COL3A1* and *PLOD2* (Fig. 3C and D). Other ECM-related genes (*FN1*, *COL1A1*, *COL4A2*, *FN1*-*EDA*, *CCN2*, *SERPINE1*) remained unchanged, indicating a selective transcriptional response. These genes are functionally linked to fibrotic tissue remodeling, and many are regulated by canonical TGF-β1/SMAD signaling.

To determine whether this transcriptional activation was accompanied by increased expression of canonical myofibroblast markers at the protein level, Western blot analysis was performed for α-SMA (encoded by *ACTA2*), TGF-β1 (encoded by *TGFB1*), and LH2 (encoded by *PLOD2*), which are key mediators of contractility, autocrine signaling and ECM stabilization, respectively. Despite the transcriptional upregulation of *ACTA2* and *TGFB1*, no significant changes in α-SMA and TGF-β1 or LH2 protein levels were detected 24 h postirradiation (Fig. 3E–H), suggesting delayed translational or posttranscriptional regulation.

Additionally, HDF migration in fibrin hydrogels was assessed (Fig. 3I). Quantification of the total cell number beyond the spheroid core revealed no significant increase in cell outgrowth when the spheroids were composed exclusively of HDFs following LDIR exposure (Fig. 3J). Interestingly, under identical conditions and even in the absence of irradiation, prevascularized dermal spheroids exhibited robust fibroblast outgrowth, as evidenced by a significant increase in the area covered by HDFs compared with spheroids composed exclusively of fibroblasts, highlighting the importance of endothelial–stromal crosstalk (Fig. 3K).

These findings indicate that LDIR initiates a partial myofibroblast-associated transcriptional program in fibroblasts; however, in the absence of endothelial signals, this response does not translate into functional migration and full phenotypic activation.

### 2.4 LDIR boosts the proangiogenic secretome of HDF-ECFC-conditioned medium and promotes *in vivo* angiogenesis

**Fig. 4:**
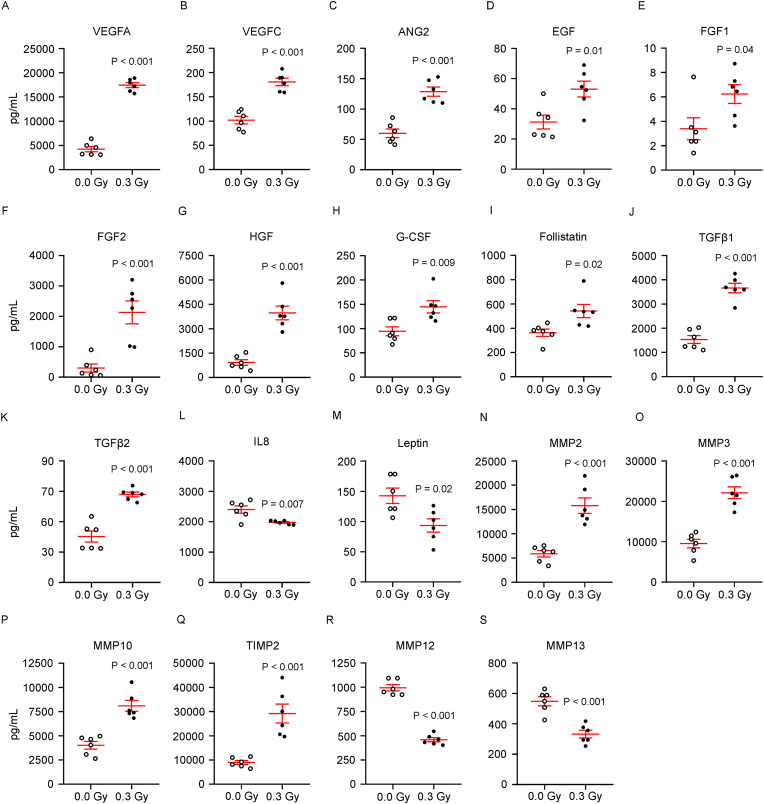
LDIR enhances the angiogenic secretome of HDF-ECFC spheroids by increasing proangiogenic and growth factor concentrations. HDFs and ECFCs were coseeded at a 5:1 ratio in agarose microwell molds to form HDF-ECFC spheroids within 24 h (day 0), followed by exposure to LDIR (0.3 Gy) or sham irradiation (0.0 Gy). Spheroids (n = 81 per condition) were transferred to a 24-well plate with 500 μL of basal medium per well. After 24 h, the conditioned medium was collected for multiplex protein analysis. Concentrations of individual factors (pg/mL) in the conditioned media from 0.0 Gy- to 0.3 Gy-exposed spheroids are shown. Individual data and the mean ± SEM (red) are presented. Statistical analysis was performed via an unpaired two-tailed *t*-test under the assumption of a normal distribution (n = 6 per group). Significant P values are indicated. (For interpretation of the references to colour in this figure legend, the reader is referred to the Web version of this article.)

To assess the impact of LDIR on the paracrine function of HDF-ECFC spheroids, conditioned media were collected 24 h postirradiation from the irradiated and sham-treated groups. Multiplex immunoassays revealed that LDIR exposure led to a significant increase in several proangiogenic cytokines and growth factors, including VEGFA, VEGFC, ANG2, EGF, FGF1, FGF2, HGF, G-CSF, Follistatin, TGFβ1 and TGFβ2. In contrast, the levels of IL-8 and leptin were significantly decreased. With respect to matrix remodeling factors, LDIR induced significant upregulation of MMP2, MMP3, MMP10, and TIMP2, whereas MMP12 and MMP13 were significantly downregulated (Fig. 4A–S). No changes were observed in the levels of endoglin, endothelin-1 (ET-1), PLGF, MMP1, MMP7, TIMP1, or TIMP4 following LDIR exposure (Supplementary Fig. 1A–G).

**Fig. 5:**
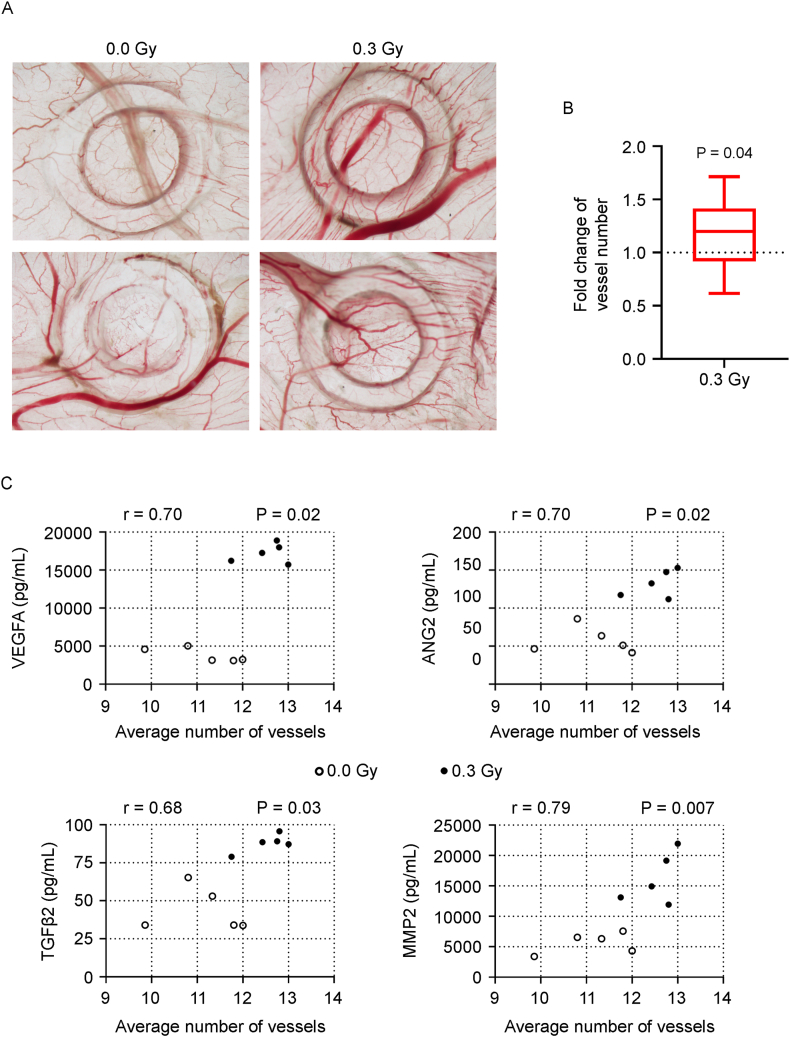
LDIR enhances *in vivo* angiogenesis through the secretome of HDF-ECFC spheroids. HDFs and ECFCs were coseeded at a 5:1 ratio in agarose microwell molds to form HDF-ECFC spheroids within 24 h (day 0), followed by exposure to LDIR (0.3 Gy) or sham irradiation (0.0 Gy). The spheroids (n = 81 per condition) were transferred to basal medium, and 24 h later, the conditioned medium was collected and used in the CAM assay. Each CAM received one sham and one LDIR-conditioned medium sample in separate silicone rings. (**A**) Representative images of the inoculation sites showing neovascularization induced by conditioned media from 0.0 Gy to 0.3 Gy conditions. (**B**) Fold change in the number of newly formed vessels induced by 0.3 Gy CM relative to 0.0 Gy CM. The data are shown as box-and-whisker plots representing the means, interquartile ranges, and minimum/maximum values. Statistical analysis was performed via a paired two-tailed *t*-test under the assumption of a normal distribution (n = 25 CAMs). Significant P values are indicated. (**C**) Correlations between the average number of recruited vessels per conditioned medium and the concentrations (pg/mL) of VEGFA, ANG2, TGF-β2, and MMP2. Pearson correlation coefficients (r) and P values are reported.

The angiogenic potential of these conditioned media was evaluated via the chorioallantoic membrane (CAM) assay. Compared with control media, media from irradiated spheroids induced significantly greater neovascularization (illustrated in Fig. 5A and quantified in Fig. 5 B). Pearson correlation analysis revealed strong positive associations between vessel number and the concentrations of VEGFA, ANG2, TGFβ2, and MMP2 across all conditioned media. Interestingly, the irradiated group presented increased levels of these molecules, which were positively associated with increased neovascularization (Fig. 5C).

These data indicate that LDIR enhances the angiogenic paracrine activity of HDF-ECFC spheroids, driving robust angiogenesis *in vivo*.

### 2.5 LDIR combined with fibrin-embedded HDF-ECFC spheroids enhances wound healing in a preclinical diabetic mouse model

**Fig. 6:**
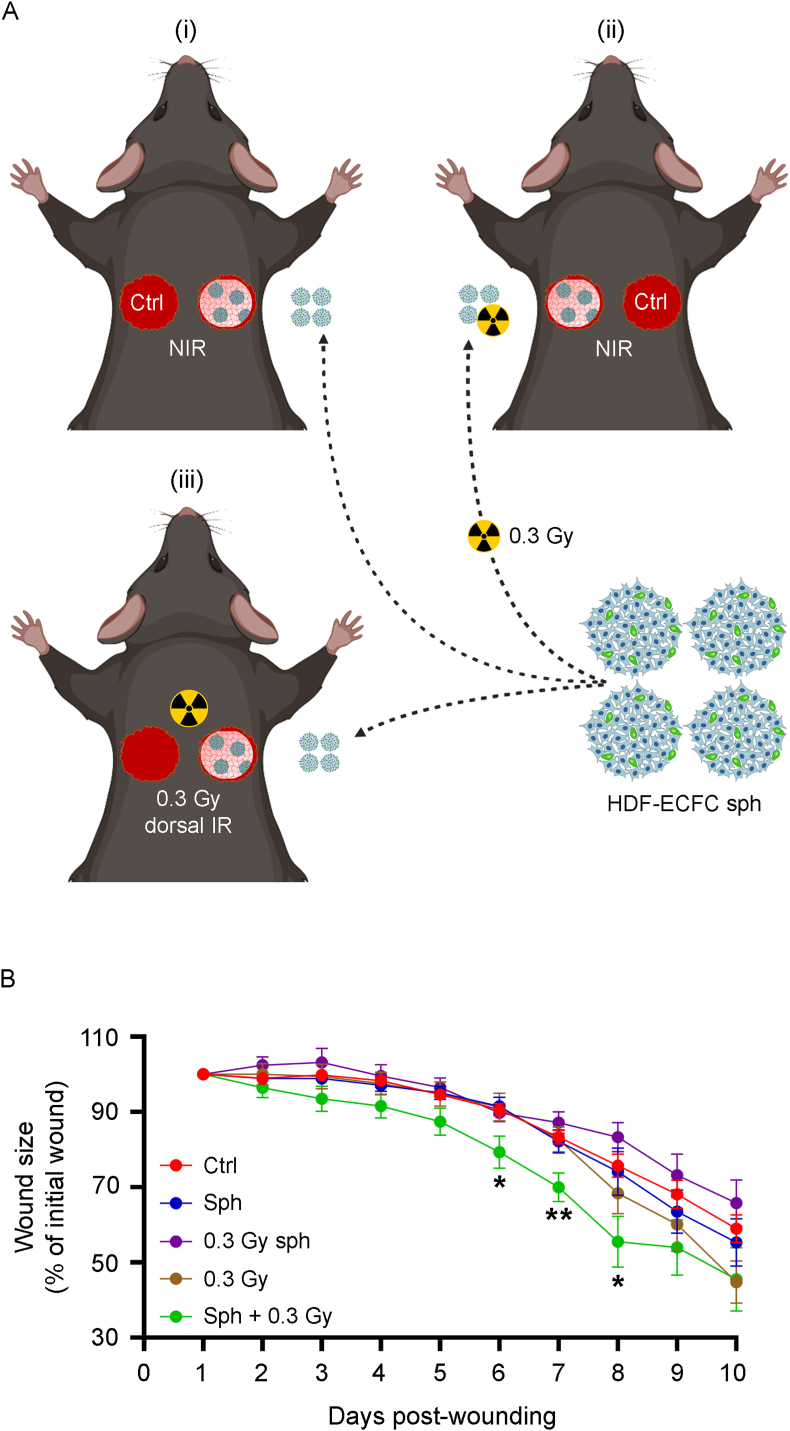
LDIR combined with fibrin-embedded HDF-ECFC spheroids accelerates wound healing in the *db/db* diabetic mouse model. (A) To assess the therapeutic potential of LDIR in combination with HDF-ECFC spheroids, full-thickness excisional wounds were created on the dorsal skin of the *db/db* diabetic mice. HDF-ECFC spheroids were embedded in a xeno-free fibrin hydrogel supplemented with aprotinin prior to application. Three experimental groups were tested: (i) nonirradiated mice receiving HDF-ECFC spheroids in one wound and vehicle in the contralateral wound (internal control); (ii) nonirradiated mice receiving preirradiated (0.3 Gy) HDF-ECFC spheroids in one wound and vehicle in the contralateral wound; and (iii) mice receiving nonirradiated HDF-ECFC spheroids followed by local *in vivo* exposure of the dorsal skin to LDIR (0.3 Gy), with the contralateral wound as a control. (**B**) Wound area was monitored over 10 days in the following groups: untreated controls (Ctrl; n = 33 wounds, days 1–7; n = 23, days 8–10), HDF-ECFC spheroids (sph; n = 21, days 1–7; n = 11, days 8–10), preirradiated HDF-ECFC spheroids (0.3 Gy sph; n = 12, days 1–10), *in vivo* 0.3 Gy only (0.3 Gy; n = 12, days 1–7; n = 6, days 8–10), and nonirradiated HDF-ECFC spheroids followed by *in vivo* exposure of the dorsal skin to 0.3 Gy (sph + 0.3 Gy; n = 12, days 1–7; n = 6, days 8–10). Wound size is presented as the mean ± SEM over time. A mixed-effects model (REML method) followed by Bonferroni correction was used for statistical analysis. Significant differences vs. untreated controls are indicated. ∗P < 0.05, ∗∗P < 0.01.

To evaluate the therapeutic potential of LDIR in combination with HDF-ECFC spheroids, full-thickness excisional wounds were created in the dorsal skin of *db/db* diabetic mice. The HDF-ECFC spheroids were embedded in a xeno-free fibrin hydrogel supplemented with aprotinin before application [29,30]. Three experimental conditions were assessed and are illustrated in Fig. 6A: (i) Nonirradiated mice in which HDF-ECFC spheroids were applied to one wound and the contralateral wound served as the control; (ii) Nonirradiated mice receiving preirradiated (0.3 Gy) HDF-ECFC spheroids in one wound, with the contralateral wound as the control; and (iii) mice in which nonirradiated HDF-ECFC spheroids were applied to one wound, with the contralateral wound as the control, followed by *in vivo* exposure of the dorsal skin (including the applied spheroids) to 0.3 Gy (hereafter referred to as HDF-ECFC sph + 0.3 Gy *in vivo*).

Wound closure was monitored over a 10-day period [31,32] (Fig. 6B). HDF-ECFC sph + 0.3 Gy *in vivo* significantly accelerated wound healing between days 6 and 8. In contrast, preirradiated spheroids did not enhance wound closure, highlighting the importance of *in vivo* irradiation. Notably, neither LDIR *per se* (in the absence of spheroids) nor nonirradiated HDF-ECFC spheroids promoted wound closure compared with the respective internal controls.

**Fig. 7:**
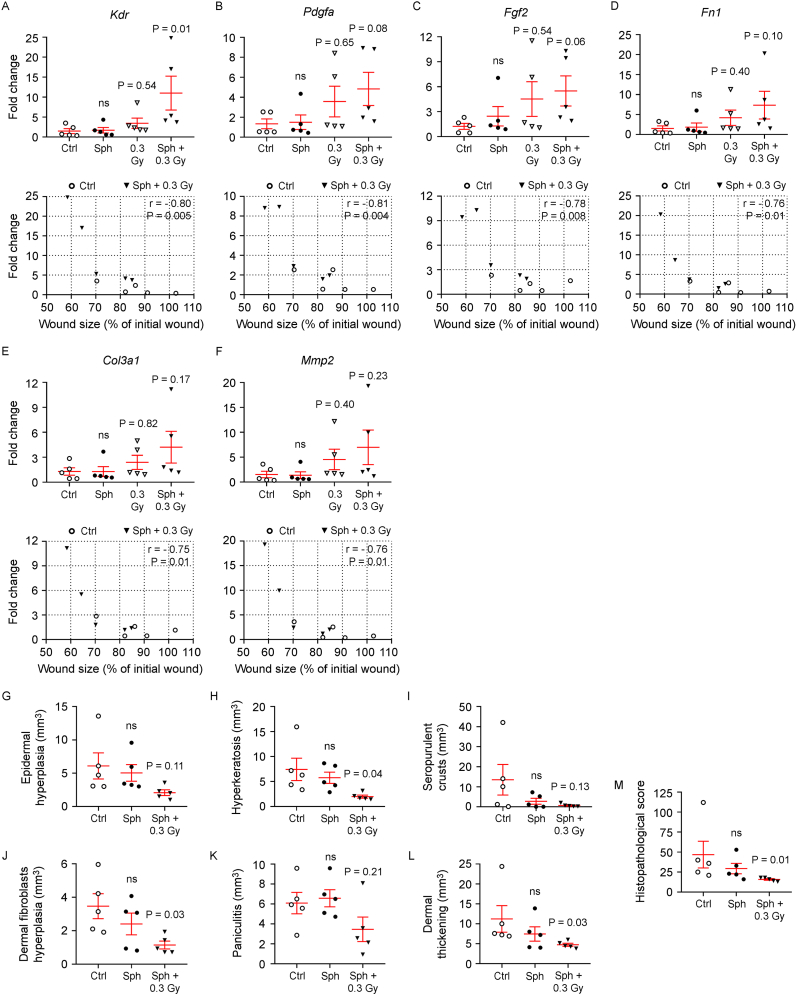
LDIR combined with fibrin-embedded HDF-ECFC spheroids enhances wound repair in the *db/db* diabetic mouse model. In the *db/db* mice, one of the following treatments was directly applied to the wound: no treatment (Ctrl), HDF-ECFC spheroids (Sph), *in vivo* LDIR exposure (0.3 Gy), or HDF-ECFC spheroids combined with *in vivo* LDIR (Sph + 0.3 Gy). On day 7 postwounding, the gene expression of (**A**) *Kdr*, (**B**) *Pdgfa*, (**C**) *Fgf2*, (**D**) *Fn1*, (**E**) *Col3a1*, and (**F**) *Mmp2* in the wound bed was quantified via qRT‒PCR (n = 5 wounds per group). Cycle threshold values were normalized to 18S values to calculate relative gene expression, presented as the fold change versus control wounds. The data are shown as individual values and means ± SEMs (in red). One-way ANOVA with Bonferroni correction was used (for normally distributed data). Lower panels: correlation analysis between the mRNA expression of each gene and wound size on day 7 was performed via Pearson correlation; *r* and P values are shown. On day 10, regenerated skin samples were collected for stereological histopathological analysis. The volume (mm^3^) of (**G**) epidermal hyperplasia, (**H**) hyperkeratosis, (**I**) seropositive crusts, (**J**) dermal fibroblast hyperplasia, (**K**) panniculitis, and (**L**) dermal thickening was quantified. (**M**) An overall histopathological score was assigned per wound, with higher scores indicating greater tissue damage. Data (n = 5 wounds per group) are shown as individual values and means ± SEMs (in red). Statistical analysis was performed via one-way ANOVA with Bonferroni correction (**G**–**K**) or the Kruskal–Wallis test with Dunn's correction (**L**–**M**), depending on the data distribution. P values for the 0.3 Gy and Sph + 0.3 Gy groups are shown; ns = not significant. (For interpretation of the references to colour in this figure legend, the reader is referred to the Web version of this article.)

On day 7, qRT‒PCR analysis of the wound tissue revealed significant upregulation of the proangiogenic gene *Kdr* in the HDF-ECFC sph + 0.3 Gy group *in vivo* (Fig. 7A, upper panel). Nonsignificant upwards trends were also observed for *Pdgfa*, *Fgf2*, *Fn1*, *Col3a1*, and *Mmp2* (Fig. 7B–F, upper panels), whereas no significant changes were detected for *Ccn2*, *Col1a1*, or *Lama1* (Supplementary Fig. 2A–C). Moreover, Pearson correlation analysis revealed that the expression levels of all genes showing significant changes or increasing trends (*Kdr*, *Pdgfa*, *Fgf2*, *Fn1*, *Col3a1*, and *Mmp2*) were significantly and inversely correlated with the residual wound area (Fig. 7A–F, lower panels), supporting a potential association with the wound-healing response.

Moreover, no significant differences in gene expression were detected across all the assessed targets in the group treated with nonirradiated HDF-ECFC spheroids compared with the control group (Fig. 7A–F). In the *in vivo* 0.3 Gy group, modest increases in *Pdgfa*, *Fgf2*, and *Mmp2* expression were detected (Fig. 7B, C, F). However, no statistically significant correlations with the wound area were detected for any of the targets in this group (Supplementary Fig. 3). Taken together, our data suggest that nonirradiated spheroids or LDIR *per se*, i.e., in the absence of spheroids, are insufficient to induce a gene expression profile strongly associated with wound closure.

Histological analysis performed on day 10 post-wounding revealed a significant reduction in the following features: epidermal hyperplasia, hyperkeratosis, crust formation, dermal fibroblast hyperplasia, and panniculitis in the HDF-ECFC spheroids + *in vivo* 0.3 Gy group (Fig. 7G–K). Compared with the other groups, this experimental group presented the lowest dermal thickness values (Fig. 7L). Illustrative images are presented in Supplementary Fig. 4. A composite skin integrity score further confirmed superior tissue organization and quality in this group (Fig. 7M). Moreover, the experimental group treated with nonirradiated HDF-ECFC spheroids showed no improvements in any histological parameter relative to the control group (Fig. 7G–M).

These findings highlight the synergistic effect of LDIR and HDF-ECFC spheroids in promoting wound resolution and improving histological features in the *db/db* diabetic mouse model.

## 3 Discussion

Chronic diabetic wounds continue to impose significant health and economic burdens, primarily due to deficient angiogenesis, impaired extracellular matrix (ECM) remodeling, and persistent inflammation [[1], [2], [3], [4],^7^]. Conventional therapies often fail to fully restore vascular networks and a durable matrix structure [4,33].

This study demonstrated that LDIR at 0.3 Gy synergistically enhances the regenerative efficacy of prevascularized dermal spheroids embedded in a xeno-free, aprotinin-stabilized fibrin hydrogel to markedly improve wound healing outcomes in the *db/db* diabetic mouse model.

LDIR stimulates endothelial activation and angiogenesis in different experimental models [[22], [23], [24], [25], [26]], findings that were further validated in human peritumoral tissues exposed to LDIR during radiotherapy [21]. Importantly, 0.3 Gy exposure increased VEGF expression and neovascularization in a hindlimb ischemia model [24], whereas exposure of adipocytes to LDIR increased the proangiogenic potential of their secretome [23]. These findings align with our observations: LDIR-treated spheroids presented enhanced endothelial sprouting, upregulated angiogenic genes (*KDR*, *PECAM1*, *CDH5*, and *COL4A2*), and elevated secretion of VEGFA, ANG2, and MMP2, resulting in robust neovascularization in the CAM assay. These results support the ability of LDIR to potentiate angiogenic activity in engineered cellular therapies, such as HDF-ECFC spheroids, for CW repair.

LDIR did not alter the morphology or viability of HDF-ECFC spheroids, as shown by the unaltered core size and ATP content. However, it significantly enhanced endothelial sprouting and fibroblast radial outgrowth in fibrin matrices. These functional enhancements were underpinned by the transcriptional upregulation of angiogenic (*KDR*, *PECAM1*, *CDH5*, and *COL4A2*) and early profibrotic genes (*ACTA2*, *TGFB1*, and *PLOD2*) and were confirmed at the protein level for α-SMA, LH2, and TGF-β1. While the upregulation of these profibrotic markers could raise concerns about pathological fibrosis, the concomitant increase observed in both spheroids and wound tissue at day 7 (*Fgf2*, *Col3a1* and *Fn1*) reflects a transient, physiological fibroblast activation essential for normal wound repair rather than fibrotic pathology. In acute wound healing, temporary increases of TGFB1, Fgf2, Col3a1, Fn1 and related markers support fibroblast proliferation, migration, and extracellular matrix deposition during the proliferative phase [34,35]. In our diabetic wound model, where fibroblast activity and granulation tissue formation are typically impaired, this early upregulation likely indicates restoration of physiological reparative processes, consistent with the accelerated wound closure observed. Importantly, histological analysis at day 10 showed reduced fibroblast hyperplasia and collagen accumulation in the HDF-ECFC spheroids + *in vivo* 0.3 Gy group compared with controls, demonstrating that early profibrotic gene activation supports organized matrix deposition without leading to excessive scarring at later time.

Moreover, irradiated HDF-ECFC spheroids secreted a distinct proangiogenic paracrine profile, including increased levels of VEGFA, FGF2, ANG2, MMP2, and TGFβ isoforms, while simultaneously decreasing molecules such as IL-8 and leptin, potent chemoattractants and mediators of prolonged inflammation. While IL-8 plays a critical role in neutrophil recruitment during the inflammatory phase of acute wounds, excessive IL-8 production in chronic wounds contributes to a prolonged and dysfunctional inflammatory response, impeding progression to the proliferative and remodeling phases [36,37]. Therefore, the observed reduction of IL-8 by LDIR-treated spheroids is consistent with a shift from a pro-inflammatory to a pro-resolving environment, potentially enhancing their therapeutic efficacy in chronic wounds. Collectively, these findings suggest that LDIR reprograms the molecular and secretory phenotypes of prevascularized dermal spheroids toward a balanced regenerative state that couples angiogenic activation with controlled inflammation, a profile particularly advantageous for chronic wound repair where excessive inflammation and impaired angiogenesis co-exist.

Another relevant contribution of this work is the assessment of the effects of LDIR on spheroids composed exclusively of fibroblasts. While LDIR induced partial transcriptional priming (upregulating *ACTA2*, *LAMA1*, and *TGFB1*), this priming was not accompanied by increased protein expression or fibroblast migration. This highlights that in the absence of endothelial cells, LDIR fails to induce functional myofibroblast activation, underscoring the importance of endothelial–stromal interactions for LDIR responsiveness.

To evaluate whether the *in vitro* priming effects of LDIR on HDF-ECFC spheroids could translate into enhanced therapeutic efficacy *in vivo*, we used the *db/db* diabetic mouse model. This model not only recapitulates key features of human type 2 diabetes mellitus, including impaired wound healing [38] but is also widely recognized for its translational relevance in testing regenerative therapies [[39], [40], [41], [42], [43]]. Our study was structured around the hypothesis that the combination of LDIR and engineered cell-based constructs would result in synergistic effects in CW repair. To test this, we assessed two experimental conditions: (i) HDF-ECFC spheroids that were preirradiated with LDIR prior to implantation to determine whether they retained enhanced regenerative properties in the wound bed; and (ii) nonirradiated HDF-ECFC spheroids applied to the wound and subsequently exposed to *in vivo* LDIR, thereby enabling simultaneous modulation of both the spheroids and the host microenvironment. These effects were compared to those of individual components alone (HDF-ECFC spheroids or LDIR) to determine whether either intervention alone was sufficient, and whether their combination provided a synergistic benefit, as previously indicated by our *in vitro* findings.

Although our *in vitro* results showed that LDIR enhances the secretory activity of HDF-ECFC spheroids, we did not evaluate the effect of conditioned medium from irradiated spheroids *in vivo* due to well-established limitations of secretome-based therapies [3,16,20]. While spheroids release high levels of pro-regenerative factors, topical application of isolated cytokines and growth factors has shown poor clinical efficacy, largely because of their rapid degradation and limited tissue penetration [3,16,20]. In contrast, cell-laden biomaterials such as hydrogel-embedded spheroids enhance *in vivo* performance by promoting cell survival, shielding against immune clearance, and providing sustained paracrine signaling, which are key elements for effective wound regeneration [3,16,20].

*In vivo*, our results from the *db/db* diabetic mouse model revealed that only the combination of HDF-ECFC spheroids and *in situ* LDIR significantly accelerated wound healing. Neither preirradiated spheroids, LDIR alone, nor nonirradiated spheroids improved the closure kinetics or histological outcomes. These findings suggest that modulation of the host niche by LDIR is essential for therapeutic efficacy. Notably, only the combined treatment upregulated *Kdr, Pdgfa, Fgf2, Fn1, Col3a1* and *Mmp2,* with their expression levels inversely correlated with the wound area, indicating that a coordinated pro-regenerative response was absent in all other conditions.

Histological analysis revealed clear improvements in the combined therapy group, including reduced epidermal hyperplasia, hyperkeratosis, dermal fibroblast hyperplasia, dermal thickening, inflammation, and overall better skin architecture, as evidenced by the lowest dermal thickness and highest composite integrity score. This effect was not observed in any other group, including those treated with nonirradiated spheroids.

A xeno-free fibrin hydrogel supplemented with aprotinin was used as a delivery matrix to support spheroid administration. According to previous studies, fibrin provides a provisional ECM that supports cell adhesion, migration, and growth factor retention, whereas aprotinin extends scaffold persistence by limiting premature fibrinolysis [12,29,30,44]. This creates a transient but pro-regenerative microenvironment at the wound interface. However, despite these favorable properties, combining the matrix with either nonirradiated or preirradiated spheroids failed to induce functional regeneration or significant histological repair. These findings underscore that local delivery of LDIR to the wound niche is essential to unlock the full regenerative potential of the therapeutic construct. Together, these findings position LDIR as a uniquely versatile tool in regenerative medicine that can prime both engineered cell constructs and the pathological wound niche to achieve synergistic healing outcomes. Importantly, the 0.3 Gy dose used here is well below the cytotoxic threshold and can be delivered via standard clinical superficial electron beam therapy, facilitating translational relevance.

From a translational perspective, both components of our therapeutic approach, LDIR and cell-laden biomaterials, have individual precedents in clinical practice that support their feasibility. LDIR is already used to modulate inflammation, immune activity, and pathological tissue remodeling in nonmalignant conditions such as osteoarthritis, tendinitis, and keloids [45], offering a clinically accepted modality that could be repurposed for wound healing. In parallel, bioengineered constructs composed of viable cells have gained regulatory approval for CWs, not as tissue replacements but as paracrine stimulators of regeneration [3]. Notably, even allogeneic fibroblast sheets have demonstrated wound closure efficacy and cell persistence comparable to those of autologous constructs, reinforcing the practicality of immunomodulatory, off-the-shelf therapies [46]. Moreover, the regenerative effects of these constructs are largely independent of cell engraftment and remain effective even when cryopreserved [3,47]. These findings highlight the importance of sustained cytokine release even when there is no structural integration [3]. In this context, our strategy, leveraging LDIR to simultaneously modulate the host niche and potentiate the putative paracrine function of embedded spheroids, could represent a clinically viable and scalable solution for treating chronic, nonhealing wounds.

Several limitations of this study warrant further investigation. First, gene expression analysis was conducted only on day 7 post-wounding, a time point selected on the basis of the observation of significant differences in wound closure across experimental groups. While this provides insight into the regenerative phase, earlier time points, particularly day 3, during peak inflammation, could provide further mechanistic insight, given the known anti-inflammatory effects of LDIR at this stage [24,27,28]. Future studies incorporating earlier transcriptional profiling are warranted to capture the full temporal dynamics of LDIR-mediated modulation. Additionally, our analysis focused on acute wound healing dynamics through day 10; thus, extended longitudinal studies are needed to evaluate scar maturation, vascular remodeling, and the biomechanical integrity of the repaired tissue [48].

While this study provides mechanistic evidence that LDIR potentiates the angiogenic and paracrine activity of spheroids, the *in vivo* requirement for host niche irradiation indicates additional, yet unidentified, host-mediated mechanisms. Future research should therefore focus on dissecting how LDIR modulates crosstalk between endothelial, stromal and immune cells in wound microenvironment, how these interactions influence angiogenesis and inflammation resolution, and whether specific cytokine signatures can predict therapeutic outcomes. Literature suggests that LDIR may influence endothelial cell activation, modulate immune and stromal cell behavior, and interact with hypoxic conditions to potentiate proangiogenic signaling [[21], [22], [23], [24], [25], [26], [27], [28]]. These hypotheses remain to be tested in the context of chronic wound repair, for instance through *in vivo* lineage tracing, conditional knockouts, or high-resolution cellular profiling to delineate specific host cell contributions. Moreover, systematic optimization of LDIR delivery parameters, including dose, timing, and fractionation schemes, is imperative to maximize therapeutic efficacy and support clinical translation.

## 4 Conclusion

This study demonstrates that LDIR reprograms engineered HDF-ECFC spheroids toward a proangiogenic and pro-regenerative phenotype, thereby amplifying their potential for chronic wound repair. Importantly, *in vivo* findings revealed that local irradiation of the wound niche is indispensable to achieve full therapeutic benefit, unveiling a previously unrecognized level of host-construct interplay. The synergistic combination of LDIR and fibrin-embedded prevascularized spheroids therefore establishes a new paradigm in chronic wound therapy, where radiation acts not merely as a priming cue for engineered constructs but as a co-therapeutic modulator of the regenerative microenvironment. This context-dependent and clinically accessible strategy offers a translationally relevant framework for restoring vascularization and tissue repair in nonhealing wounds.

## 5 Materials and methods

### 5.1 Cell culture

Human umbilical cord blood was obtained from healthy donors following a protocol approved by the Ethics Committee of CHUSJ (Centro Hospitalar Universitário de São João, Porto, Portugal), as previously described [49]. Endothelial colony forming cells (ECFC) were isolated from umbilical cord blood using established protocols [[50], [51], [52]]. Human umbilical cord blood (50–80 cc) was obtained and used within 12 h of collection. Blood diluted 1:1 with Hanks' balanced salt solution (HBSS; Sigma) was layered over an equivalent volume of Histopaque 1077 (Sigma) and centrifuged for 30 min at room temperature. The resultant cord blood mononuclear cell (CBMNC) fraction was collected and treated with red blood cell lysis buffer (eBioscience). The CBMNCs were then cultured on type I collagen-coated tissue culture plates (BD Biosciences) with EGM-2MV medium (Lonza) supplemented with 10 % FBS. After 36h, nonadherent cells were removed and the medium was changed daily for adherent cells until the first passage. Colonies of ECFCs appeared between 7 and 21 days of culture. Once a colony grew to the size of a 5x field of view, the cells were detached with 0.05 % Trypsin-EDTA (Life Technologies) and plated onto tissue culture-treated 6-well plates (Beckton, Dickson and Company (BD)) for continued culture in EGM-2MV. When the cells reached ∼80 % confluency, they were again detached with 0.05 % Trypsin-EDTA and plated onto 25-cm^2^ tissue culture flasks for the first passage (P1) or 75-cm^2^ tissue culture flasks for subsequent passages, until frozen. Phenotypic expression and functional parameters were characterized as described in Ref. [53]. All blood donors were kept anonymous, so the need for written consent was waived. ECFCs were used between passages 6 and 10. The cells were expanded in MCDB 131 medium supplemented with 5 % Fetal Bovine Serum (FBS, Biowest), 1 % penicillin/streptomycin, 2 mM L-glutamine, 1 μg/mL ascorbic acid, 0.2 μg/mL hydrocortisone, and the following growth factors were used: 2 ng/mL VEGF, 20 ng/mL IGF-1R, 10 ng/mL FGF, and 5 ng/mL EGF. The supplements were reconstituted in 0.1 % human serum albumin in phosphate-buffered saline (PBS, pH 7.4). Normal Human Neonatal Dermal Fibroblasts (HDFs) were commercially obtained (Lonza, CC-2509) and used between passages 5 and 10. The cells were cultured in Dulbecco's Modified Eagle Medium (DMEM, GIBCO) supplemented with 10 % FBS (GIBCO) and 1 % penicillin/streptomycin (GIBCO). HDFs were maintained in 10 cm tissue culture dishes at 37 °C in a humidified incubator with 5 % CO_2_. The cells were maintained in 10 cm plates at 37 °C in a 5 % CO_2_ humidified incubator. ECFCs were passaged at 70–80 % confluence, and HDFs were passaged at 80–90 % confluence. The media were changed every 2 days (ECFCs) or 4 days (HDFs). For *in vitro* studies, HDF spheroids were cultured in HDF medium, while coculture spheroids were maintained in ECFC medium. For *in vivo* experiments, spheroids were cultured in xeno-free ECFC medium similar to the standard but without FBS and with the VEGF concentration increased to 10 ng/mL, as previously optimized [52]. Plastem (Grifols) was used as a serum substitute, reconstituted in MCDB 131 and filtered through a 0.2 μm membrane.

### 5.2 Spheroid generation

Spheroids were generated via MicroTissues® 3D Petri Dish® technology (catalogue: #12–81). Agarose micromolds (2 % agarose in 0.9 % NaCl) were prepared via commercial templates and placed in 12-well tissue culture plates. After equilibration with culture medium, the molds were transferred to cell culture plates for seeding. HDF spheroids were generated by seeding 10^6^ HDF cells in 200 μL of HDF medium per mold. For coculture spheroids, HDFs and ECFCs were resuspended in ECFC medium at a 5:1 ratio and seeded at 12,000 cells per spheroid (10,000 HDFs and 2000 ECFCs). The mixture of HDFs and ECFCs was allowed to settle in the MicroTissues® 3D Petri Dish® molds for 45 min before 25 mL of medium was added to the 10 cm culture plate containing the molds, as required to accommodate the acrylic phantom used for LDIR treatment. Spheroids formed within 24 h of spontaneous cell aggregation in the microwells (considered the day of maturation). Quantitative analysis of multiple spheroids showed a coefficient variation of only 4 % in cross-sectional area (n = 58), confirming their uniform size and shape.

### 5.3 Spheroid mold irradiation

Spheroid-containing plates were subjected to CT simulation via a helical scanner (Somatom Sensation, Siemens) with 2 mm slice thickness and 1 mm axial reconstruction. The plates were fully filled with basal medium, and a virtual 3D treatment plan was designed using the XiO planning system (Elekta), applying a superposition algorithm and heterogeneity correction for accurate dose mapping. On day 0 of maturation, the culture medium was removed and the samples were stored at 37 °C. The plates were positioned on a phantom at the linear accelerator (Elekta Synergy S) and aligned via the light field and isocenter lasers. The plates were replenished with basal MCDB 131 or DMEM and irradiated with a 0.3 Gy dose according to the manufacturer's instructions. Sham-irradiated controls (0.0 Gy) underwent identical procedures without beam exposure. After irradiation, the stored medium was reintroduced, and the plates were incubated at 37 °C until further analysis.

### 5.4 *In vitro* evaluation of irradiated spheroids

#### 5.4.1 Spheroid size measurement

On day 1 postirradiation, brightfield images of the spheroids were acquired via a Zeiss Axio Zoom. V16 stereomicroscope. The projected area of each spheroid was measured via ImageJ 1.50i software.

#### 5.4.2 Spheroid metabolic activity

On day 1 postirradiation, spheroids (n = 8–10 per well) were transferred to a black 96-well plate and incubated with CellTiter-Glo® 3D reagent (Promega) following the manufacturer's instructions. Luminescence was measured via a TECAN Infinite M200 plate reader (1 s integration/well) and normalized to the number of spheroids.

#### 5.4.3 mRNA expression by qRT‒PCR

On day 1 postirradiation, individual spheroids were harvested from the microwells for RNA extraction via the RNeasy® Micro Kit (QIAGEN). cDNA synthesis and one round of preamplification were performed via the RT2 Nano PreAMP cDNA synthesis kit (QIAGEN). Quantitative PCR was conducted via SYBR Green chemistry (Power SYBR® Green, Invitrogen) on a 7500/7500 Fast Real-Time PCR System (Applied Biosystems). The housekeeping gene was 18S rRNA.

The PCR conditions were as follows: 10 min at 95 °C, followed by 50 cycles of 15 s at 95 °C and 1 min at 60 °C. Relative expression was calculated via the 2^(-ΔΔCt) method, with ΔCt = Ct (gene of interest) – Ct(18S) and ΔΔCt relative to the sham-irradiated group. The results are shown as the fold change (2^(-ΔΔCt)). The following genes were analyzed via probe sets from Integrated DNA Technologies: (FN1-Hs.PT.58.40986315; COL1A1- Hs.PT.58.1551779; PECAM1-Hs.PT.58.19487865; CDH5-Hs.PT.58.4732035; COL4A2-Hs.PT.58.427878).

The gene-specific primers used were as follows (5′–3′):

FN1 ED-A_F CATTGATCGCCCTAAAGGACTG.

FN1 ED-A_R TCACCCTGTACCTGGAAACT.

ACTA2_F GGGACATCAAGGAGAAACTG.

ACTA2_R CAGGCAACTCGTAACTCTTC.

KDR_F ATTCCTCCCCCGCATCA.

KDR_R GCTCGTTGGCGCACTCTT.

COL3A1_F CTGGCATTCCTTCGACTTCT.

COL3A1_R AGCTTCAGGGCCTTCTTTAC.

SERPINE1_F CAGATGAGGACAGAGTGGTTTC.

SERPINE1_R CTGTGAGTCACCCTGTAATTGG.

PLOD2_F CCCTTTGTCTCGCTCCTTATT.

PLOD2_R AGCGGGATAGAGGGAAGTTA.

CCN2_F CATTCTCCAGCCATCAAGAGAC.

CCN2_R CCACAAGCTGTCCAGTCTAATC.

TGFB1_F CGTGGAGCTGTACCAGAAATAC.

TGFB1_R CACAACTCCGGTGACATCAA.

LAMA1_F GTACAGCGTGGCCTTCTATT.

LAMA1_R GTTCCTGTCTCACTCCATTCTC.

18S_F GCCCTATCAACTTTCGATGGTAGT.

18S_R CCGGAATCGAACCCTGATT.

### 5.5 Embedding of spheroids in fibrin hydrogel

Fibrin (Fb) hydrogels were prepared at a final fibrinogen concentration previously optimized for endothelial sprouting and tubulogenesis [11]. Fibrinogen (10 mg/mL in 0.9 % NaCl), aprotinin (0.5 mg/mL in PBS), and basal MCDB 131 were mixed and polymerized by adding thrombin (16.7 U/mL in PBS). The final concentrations in the gel were 1.5 mg/mL fibrinogen, 25.2 μg/mL aprotinin, and 0.91 U/mL thrombin. Spheroids were collected on day 1 postirradiation and embedded in Fb hydrogels in ibidi chambers. The first fibrin layer was polymerized at 37 °C for 30 min to form the base. The spheroid-containing fibrin mixture was then added on top and polymerized under the same conditions, resulting in a continuous two-layered fibrin hydrogel embedding the spheroids. Each irradiated spheroid was paired with a sham-irradiated control. The constructs were cultured in their respective stored culture media.

### 5.6 Immunofluorescence analysis of fibrin hydrogels

Fb-embedded spheroids were analyzed 72 h post-embedding. The gels were washed with PBS and fixed in 4 % (w/v) paraformaldehyde for 30 min at room temperature.

For migration analysis, brightfield images of Fb-embedded HDF-ECFC spheroids were acquired via a Zeiss Cell Discoverer 7 microscope. The area occupied by migrating HDFs was measured via ImageJ 1.50i.

For immunofluorescence, the gels were permeabilized with 0.25 % (v/v) Triton X-100 for 20 min, washed twice with 0.05 % (v/v) Tween-20 in PBS, and blocked in 2 % (w/v) BSA for 1 h. The samples were incubated with rhodamine-labelled Ulex europaeus agglutinin I (UEA I, Vector Laboratories) or a FITC-conjugated anti-human vimentin antibody (Miltenyi Biotec) diluted in 1 % BSA for 1 h at room temperature followed by an overnight incubation at 4 °C. After washing, the nuclei were counterstained with DAPI for 4 h at room temperature. All steps were performed under agitation.

Images were acquired via a Zeiss Cell Observer SD spinning disk confocal microscope with a 40 × water-immersion objective for HDF-ECFC spheroids and a 10 × objective for spheroids composed exclusively of HDFs.

### 5.7 Western blot analysis

The spheroids were irradiated on day 0 of maturation and collected 24 h later. The spheroids were washed twice in cold PBS and lysed in 50 μL of modified RIPA buffer (50 mM Tris-HCl pH 7.4, 150 mM NaCl, 1 % NP-40, 0.25 % sodium deoxycholate, and 1 mM NaF) supplemented with protease inhibitors (Roche) and vanadate (phosphatase inhibitor, Roche). The samples were incubated on ice for 45 min, vortexed every 5 min, and centrifuged at 12,000×*g* for 20 min at 4 °C. The protein concentration was determined via a BCA protein assay kit (Thermo Scientific). Equal amounts of protein were resolved by SDS‒PAGE, transferred to nitrocellulose membranes (Amersham), and blocked in 3 % BSA in PBS-T (0.1 % Tween-20). The membranes were probed with primary antibodies against ACTA, TGFB1, LH2 and GAPDH, all of which were purchased from Abcam.

### 5.8 Collection of spheroid-conditioned medium

HDF-ECFC spheroids were irradiated on day 0 of maturation and collected 20 min postirradiation. For each condition, 81 spheroids were transferred to a well of a 24-well plate and incubated in 500 μL of basal MCDB 131 medium for 24 h. The conditioned medium (CM) was then collected and centrifuged at 1200 rpm for 5 min to remove debris and stored for further analysis.

### 5.9 Multiplex analysis of conditioned medium

The quantification of secreted factors in the spheroid CM was performed by Eve Technologies (Calgary, Alberta, Canada) via Luminex® xMAP® technology and the Luminex® 200™ platform (Luminex Corporation/DiaSorin) with Bio-Plex Manager™ software (Bio-Rad). The following multiplex panels were used: Human Angiogenesis & Growth Factor 17-Plex Discovery Assay® Array (HDAGP17; Cat. #HAGP1MAG-12K, MilliporeSigma); TGFβ 3-Plex Discovery Assay® Multi-Species Array (TGFβ1–3; Cat. #TGFBMAG-64K-03, MilliporeSigma); Human MMP Premixed Magnetic Luminex® Performance Assay (Cat. #FCSTM07, R&D Systems); and Human TIMP Luminex® Performance Assay 4-Plex Fixed Panel (Cat. #LKTM003, R&D Systems).

### 5.10 Chorioallantoic membrane (CAM) assay

The angiogenic potential of HDF-ECFC spheroid-conditioned media was evaluated via the chick embryo CAM assay. The fertilized eggs were windowed at embryonic development day (EDD) 3 to allow detachment of the CAM from the shell. At EDD10, sterile silicone rings were placed on the CAM, and 50 μL of CM from either irradiated or sham-irradiated spheroids was inoculated into separate rings at least 1 cm apart. After 3 days (EDD13), CAMs were fixed in 10 % neutral-buffered formalin, excised, and photographed ex ovo. The number of newly formed vessels converging toward each ring was quantified from digital images, as described previously [23].

### 5.11 Diabetic excisional wound healing model

All animal procedures complied with Directive 2010/63/EU and were approved by the institutional Animal Welfare Body (license no. 0421/000/000/2021, DGAV, Portugal). BKS. Cg-Dock7m +/+ Leprdb/J (BKS db/db) mice, aged 12–13 weeks (Charles River, Italy), were housed in groups (n = 3) under a 14 h light/10 h dark cycle with ad libitum access to food and water. After wounding, the animals were housed individually and randomized into treatment groups. Under isoflurane anesthesia (induction: 3–4 %; maintenance: 1–2 % in O_2_), the dorsal hair was removed with clippers followed by depilatory cream. Preemptive analgesia (buprenorphine, 0.1 mg/kg, subcutaneous) was administered. The dorsal skin was cleansed with sterile saline, and two full-thickness excisional wounds (6 mm in diameter, including the panniculus carnosus) were created bilaterally in the midline via a biopsy punch. Postoperative analgesia was maintained with oral buprenorphine (∼0.009 mg/mL in water) for at least 96 h. The wound area was monitored daily for 10 days via acetate tracing [31,32].

### 5.12 Administration of spheroids embedded in fibrin hydrogel

Fibrin hydrogels were prepared immediately prior to application. A fibrinogen solution (10 mg/mL in 0.9 % NaCl) was mixed with aprotinin (0.5 mg/mL in PBS) and basal MCDB 131 medium to obtain the fibrinogen–aprotinin mixture. Fifty spheroids were resuspended in 50 μL of this mixture, and thrombin (16.7 U/mL in PBS) was then added to initiate gelation. The final concentrations in the fibrin hydrogel were 1.5 mg/mL fibrinogen, 25.2 μg/mL aprotinin, and 0.91 U/mL thrombin. The spheroid-containing fibrin mixture was applied directly into the wound bed before complete gelation, allowing *in situ* polymerization within seconds under continued anesthesia and ensuring spheroid retention at the wound site.

### 5.13 Mouse irradiation procedure

Dosimetric planning was performed via a 3D treatment planning system (Monaco, Elekta). A linear accelerator (Elekta Synergy S) delivered a 0.3 Gy dose of 9 MeV electron beam radiation at room temperature. The mice were positioned in an acrylic phantom and dorsally covered with a 2 cm water-equivalent bolus to ensure uniform dose distribution. A CT scan (Somatom Sensation, Siemens) with 2 mm slices was used to delineate the planning target volume (wound area). Irradiation was performed as a single dose 24 h post-wounding. The control animals underwent identical procedures without irradiation (0.0 Gy). Anesthesia during irradiation consisted of intraperitoneal medetomidine (0.75 mg/kg) and ketamine (1 mg/kg); reversal was achieved with atipamezole (1 mg/kg).

### 5.14 Animal sacrifice and wound tissue collection

The animals were sacrificed by cervical dislocation at either day 7 or day 10 post-wounding for molecular and histological analysis, respectively. For gene expression, each wound was excised individually and minced, along with a 2 mm margin of adjacent healthy skin. For histology, the entire dorsal skin containing both wounds was harvested and fixed in 10 % neutral-buffered formalin for ≥48 h before processing.

### 5.15 Wound tissue RNA extraction and gene expression analysis via qRT‒PCR

The minced wound tissue was placed in lysing matrix tubes (1.4 mm ceramic beads, MP Biomedicals) with 350 μL of RLT buffer (QIAGEN) and homogenized in a bead beater (2 × 45 s cycles). The lysates were centrifuged at maximum speed for 3 min, and RNA was extracted via the RNeasy® Micro Kit (QIAGEN). cDNA synthesis and one round of preamplification were performed via the RT^2^ Nano PreAMP cDNA Synthesis Kit (QIAGEN). Quantitative real-time PCR was conducted via Power SYBR® Green Master Mix (Invitrogen) on a 7500/7500 Fast Real-Time PCR System (Applied Biosystems). The housekeeping gene was 18S. The cycling conditions were 95 °C for 10 min, followed by 50 cycles of 95 °C for 15 s and 60 °C for 1 min. Relative expression was calculated via the 2^−ΔΔCt method, with the untreated group as the calibrator, where ΔCt = Ct (gene of interest) – Ct (housekeeping gene) and ΔΔCt = ΔCt (sample) – ΔCt (control average). The data for each spheroid are presented as the fold change (2^(−ΔΔCt)). The primer sequences (5′–3′) used were as follows:

CCN2_F TTGGCCCAGACCCAACTAT.

CCN2_R GAGATGCCCATCCCACAG.

COL1A1_F CAGGGAATGCCTGGTGAA.

COL1A1_R TCCCAAAGGTGCTGATGG.

COL3A1_F ATGGATCTCCTGGTGGCAA.

COL3A1_R CAGGTGGGCCTGGATGA.

FGF2_F TGAAACGAACTGGGCAGTATAA.

FGF2_R GCTCTTAGCAGACATTGGAAGA.

FN1_F TCTGCACAACCAATGAAGGG.

FN1_R ACACACGTGCACCTCATC.

LAMA1_F CTGTGATCGCTGCAAGCC.

LAMA1_R AAGCAGAAGCACTCGGA.

MMP2_F CCGTGGTGAGATCTTCTTCTTC.

MMP2_R GAGCTCAGGCCAGAATGTG.

PDGFa_F CAAGACCAGGACGGTCATTTA.

PDGFa_R GGGCCAGATCAGGAAGTTG.

VEGFR2_F CGACATAGCCTCCACTGTTTAT.

VEGFR2_R TGTTCTTGTTCTCGGTGATGT.

### 5.16 Histological analysis of regenerated dorsal skin

Fixed dorsal skin was paraffin-embedded and serially sectioned at 999 μm intervals with a random start (random number 1–333, generated via app). From each selected interval, two consecutive 3 μm sections were collected for hematoxylin and eosin (H&E) staining. A minimum of 10 sections per wound were analyzed. Tissue volumes were estimated via the Cavalieri principle: (V = T × a/p × ∑Pi, where T is the section interval, a/p is the area per point, and Pi is the number of points hitting the region of interest. The digital slides were scanned via a Hamamatsu NanoZoomer system and analyzed via Visiopharm stereology software. A 6 × 6 point grid (36 points) was applied to 10 × fields of view, covering 10 % of the total tissue area per wound.

### 5.17 Statistical analysis

The data are presented as the means ± standard errors of the means (SEMs). Statistical analyses were performed via GraphPad Prism 8 (GraphPad Software). Normality was assessed via the Shapiro–Wilk test. For comparisons between two groups, unpaired or paired two-tailed t tests were used for normally distributed data, and Mann–Whitney tests were used for nonnormally distributed data. For comparisons among three or more groups, one-way ANOVA followed by Bonferroni correction was applied when normality was confirmed; otherwise, Kruskal–Wallis tests with Dunn's post hoc correction were used. Repeated measures of wound closure over 10 days were analyzed via a mixed-effects model with restricted maximum likelihood estimation and Bonferroni correction for multiple comparisons. P values < 0.05 were considered statistically significant.

## CRediT authorship contribution statement

**Filipe Rocha:** Writing – review & editing, Visualization, Validation, Methodology, Investigation, Formal analysis. **Inês Sofia Vala:** Writing – review & editing, Validation, Investigation. **Paula de Oliveira:** Writing – review & editing, Investigation. **Pedro Faísca:** Writing – review & editing, Methodology, Investigation. **Carolina Fernandes:** Writing – review & editing, Investigation. **Marta Teixeira Pinto:** Writing – review & editing, Methodology, Investigation. **Filomena Pina:** Writing – review & editing, Resources, Conceptualization. **Esmeralda Poli:** Writing – review & editing, Resources, Methodology, Conceptualization. **Isabel Diegues:** Writing – review & editing, Resources, Methodology, Conceptualization. **Eugénia Carvalho:** Writing – review & editing, Methodology, Funding acquisition, Conceptualization. **Cristina C. Barrias:** Writing – review & editing, Resources, Funding acquisition, Conceptualization. **Susana Constantino Rosa Santos:** Writing – original draft, Validation, Supervision, Resources, Project administration, Investigation, Funding acquisition, Conceptualization.

## Declaration of generative AI and AI-assisted technologies in the writing process

No Artificial intelligence (AI)-assisted technologies were used in the making of this work.

## Funding sources

This work was supported by the Fundaç ão para a Ciência e a Tecnologia (10.13039/501100001871FCT), under grant number 2022.10387. PTDC and UID 00306 - Centro Cardiovascular da Universidade de Lisboa, as well as UIDB/04539/2020, UIDP/04539/2020 and LA/P/0058/2020.

## Declaration of competing interest

The authors declare the following financial interests/personal relationships which may be considered as potential competing interests:Given their role as Managing Editor, Cristina C. Barrias had no involvement in the peer-review of this article and has no access to information regarding its peer-review. Full responsibility for the editorial process for this article was delegated to another journal editor. If there are other authors, they declare that they have no known competing financial interests or personal relationships that could have appeared to influence the work reported in this paper.

## Data Availability

All data needed to evaluate the conclusions in the paper are present in the paper or the Supplementary Materials. Any additional information required is available from the corresponding author upon request.

## Abbreviations

**3D**: Three-dimensional
**α-SMA**: Alpha-smooth muscle actin
**ACTA2**: Actin alpha 2 (gene encoding α-SMA)
**ANG2**: Angiopoietin-2
**CAM**: Chorioallantoic membrane
**CCN2**: Cellular communication network factor 2
**CDH5**: Cadherin 5 (VE-cadherin)
**COL1A1**: Collagen type I alpha 1 chain
**COL3A1**: Collagen type III alpha 1 chain
**COL4A2**: Collagen type IV alpha 2 chain
**CW**: Chronic wound
**ECFC**: Endothelial colony-forming cells
**ECM**: Extracellular matrix
**EGF**: Epidermal growth factor
**ET-1**: Endothelin-1
**FBS**: Fetal Bovine Serum
**FCT**: Fundação para a Ciência e Tecnologia
**FGF1/FGF2**: Fibroblast growth factor 1/2
**FN1**: Fibronectin 1
**FN1-EDA**: Fibronectin 1 extra domain A isoform
**G-CSF**: Granulocyte-colony stimulating factor
**GAPDH**: Glyceraldehyde-3-phosphate dehydrogenase
**Gy**: Gray (unit of absorbed radiation dose)
**HDFs**: Human dermal fibroblasts
**HDF-ECFC**: Human dermal fibroblast–endothelial colony-forming cells
**HIF**: Hypoxia-inducible factor
**HUVEC**: Human umbilical vein endothelial cells
**IL-8**: Interleukin-8
**KDR**: Vascular Endothelial Growth Factor Receptor 2
**LAMA1**: Laminin subunit alpha-1
**LDIR**: Low-dose ionizing radiation
**LH2**: Lysyl hydroxylase 2 (encoded by PLOD2)
**MMP2**: Matrix metalloproteinase-2
**MMP**: Matrix metalloproteinase
**PAI-1**: Plasminogen activator inhibitor-1 (SERPINE1)
**PDGFA**: Platelet-derived growth factor subunit A
**PECAM1**: Platelet and endothelial cell adhesion molecule 1 (CD31)
**PLGF**: Placental growth factor
**PLOD2**: Procollagen-lysine 2-oxoglutarate 5-dioxygenase 2
**qRT‒PCR**: Quantitative reverse transcription polymerase chain reaction
**SERPINE1**: Plasminogen Activator Inhibitor 1
**SDF-1α**: Stromal cell-derived factor 1 alpha
**TGF-β1/TGFβ1**: Transforming growth factor beta 1
**TGFβ2**: Transforming growth factor beta 2
**TIMP**: Tissue inhibitor of metalloproteinases
**VE-cadherin**: Vascular endothelial cadherin
**VEGFA/VEGFC**: Vascular endothelial growth factor A/C
**VEGFR2**: Vascular endothelial growth factor receptor 2 (KDR)
