# Supplementary material for: Low-dose ionizing radiation in vivo unlocks the therapeutic potential of prevascularized dermal spheroids in chronic wounds
Source: Mater Today Bio. 2025 Nov 15;35:102561. doi: 10.1016/j.mtbio.2025.102561 (PMC12676115; doi:10.1016/j.mtbio.2025.102561)
Supplement: Multimedia component 1 [file mmc1.docx]

Low-dose ionizing radiation *in vivo* unlocks the therapeutic potential of prevascularized dermal spheroids in chronic wounds

Filipe Rocha ^a^, Inês Sofia Vala ^a^, Paula de Oliveira ^a^, Pedro Faísca ^b^, Carolina Fernandes^a^, Marta Teixeira Pinto ^c^, Filomena Pina ^d^, Esmeralda Poli ^d^, Isabel Diegues ^d^, Eugénia de Carvalho ^e^, Cristina C. Barrias ^c,f,g^ and Susana Constantino Rosa Santos ^a,^*

**Supplementary Figures**


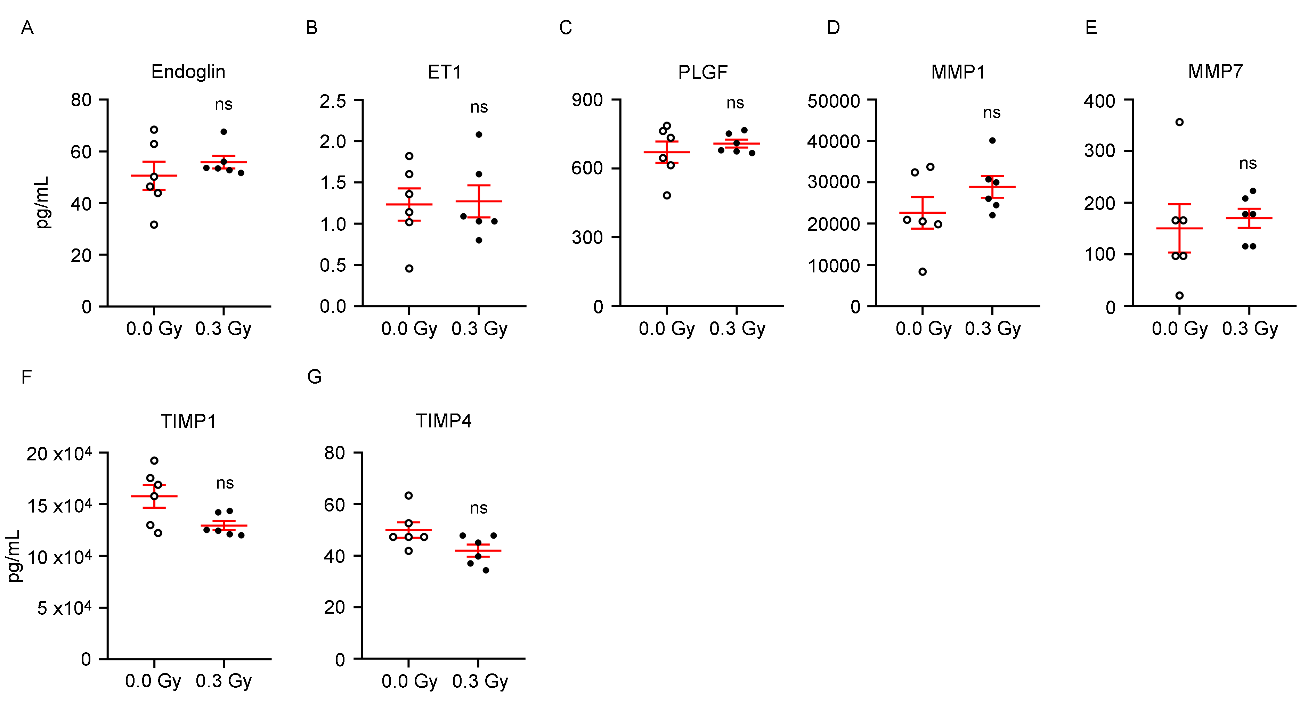


**Supplementary Fig. 1. LDIR does not alter the secretion of selected proangiogenic factors, growth factors, or ECM-regulating enzymes by HDF-ECFC spheroids.** HDFs and ECFCs were coseeded at a 5:1 ratio in agarose microwell moulds to form HDF-ECFC spheroids within 24 h (day 0), followed by exposure to LDIR (0.3 Gy) or sham irradiation (0.0 Gy). Spheroids (n = 81 per condition) were transferred to a 24-well plate with 500 μL of basal medium per well. Concentrations (pg/mL) of the indicated factors in the conditioned media of 0.0 Gy- or 0.3 Gy-exposed spheroids. Individual values and means ± SEMs (in red) are shown. The data were assumed to be normally distributed and were analysed via an unpaired two-tailed t-test (n = 6 conditioned media per group); ns=not significant.


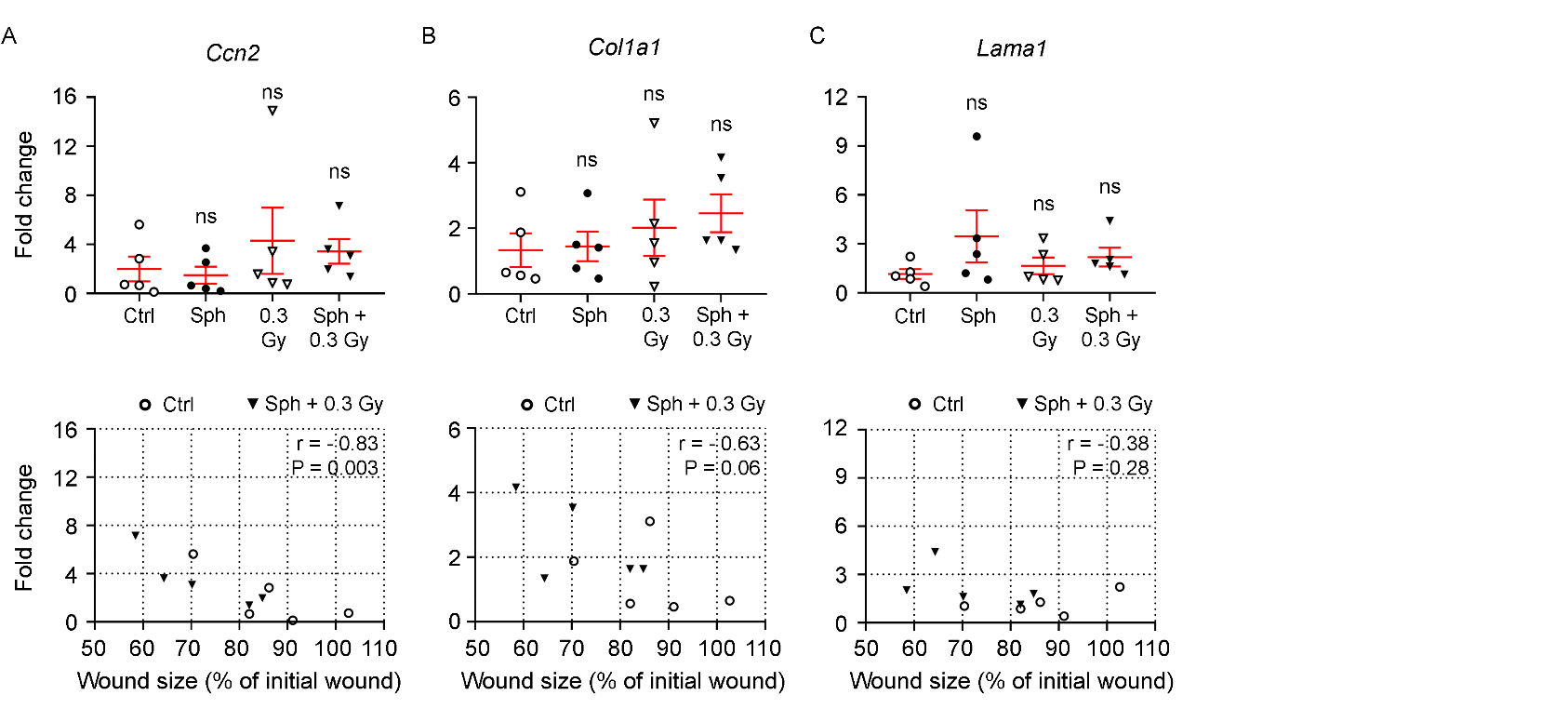


Supplementary Fig. 2. **The expression of** Ccn2**,** Col1a1 **and** Lama1 **in the wound bed remained unchanged following treatment with LDIR combined with fibrin-embedded HDF-ECFC spheroids.** In a diabetic wound model, one of the following treatments was directly applied to the wound: no treatment (Ctrl), HDF-ECFC spheroids (Sph), *in vivo* LDIR exposure (0.3 Gy), or HDF-ECFC spheroids combined with *in vivo* LDIR (Sph + 0.3 Gy). On day 7 postwounding, the mRNA expression of (**A**) Ccn2, (**B**) Col1a1 and (**C**) Lama1 in the wound bed was quantified via qRT‒PCR (n = 5 wounds per group). Cycle threshold values were normalized to 18S values to calculate relative gene expression, presented as the fold change versus control wounds. The data are shown as individual values and means ± SEMs (in red). Statistical analysis was performed via one-way ANOVA with Bonferroni correction (**A**, **C**) or the Kruskal–Wallis test with Dunn’s correction (**B**), depending on the data distribution. Lower panels: correlation analysis between the mRNA expression of each gene and wound size on day 7 was performed via Pearson correlation; r and P values are shown; ns=not significant.


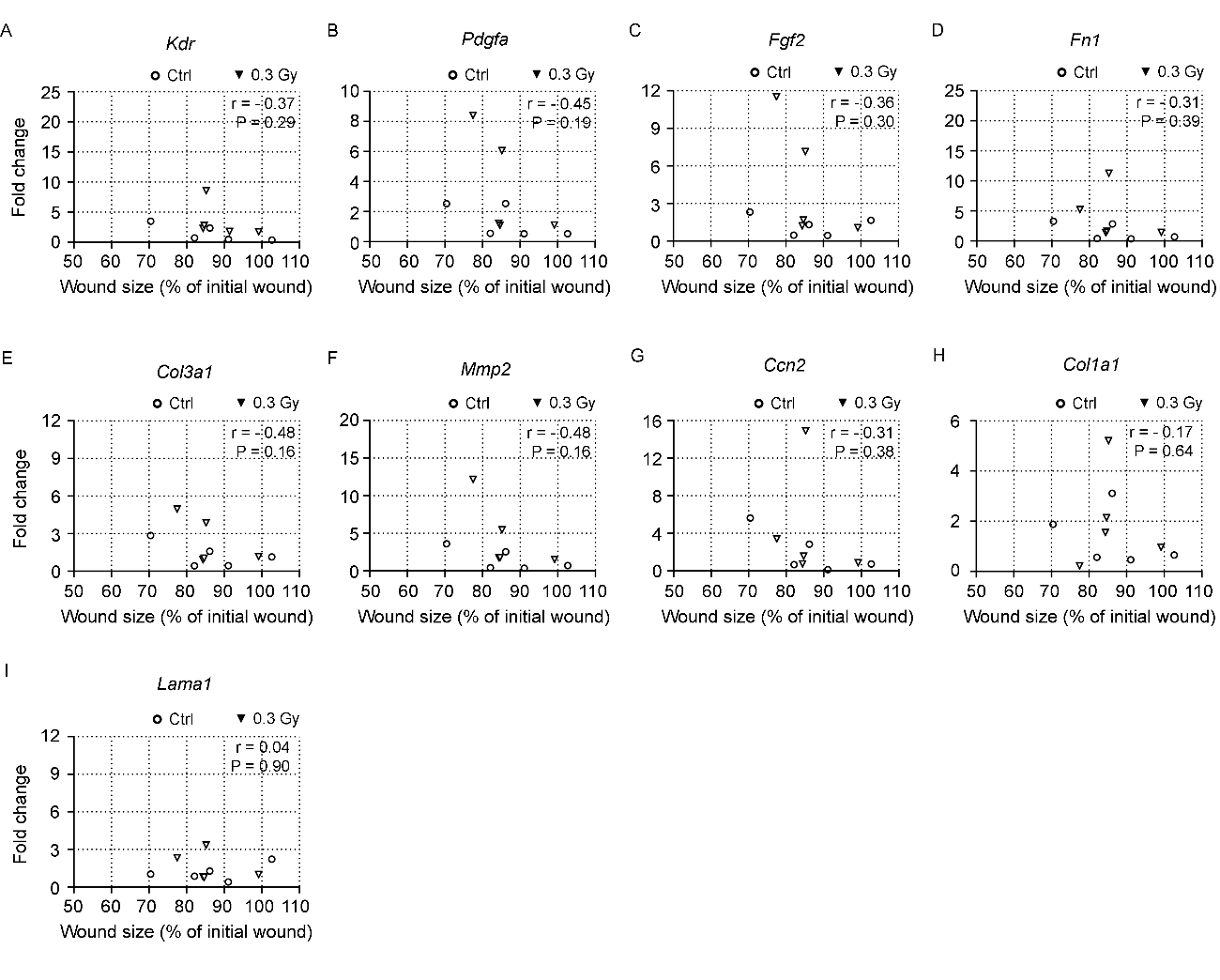


**Supplementary Fig. 3. Wound bed gene expression does not correlate with wound size following *in vivo* LDIR.** In the *db/db* diabetic model, mice received either no treatment (Ctrl) or *in vivo* exposure to LDIR (0.3 Gy). On day 7 postwounding, a correlation analysis was performed between the mRNA expression of the indicated genes in the wound bed and the size of the same animal. Pearson correlation was used; r and P values are displayed.


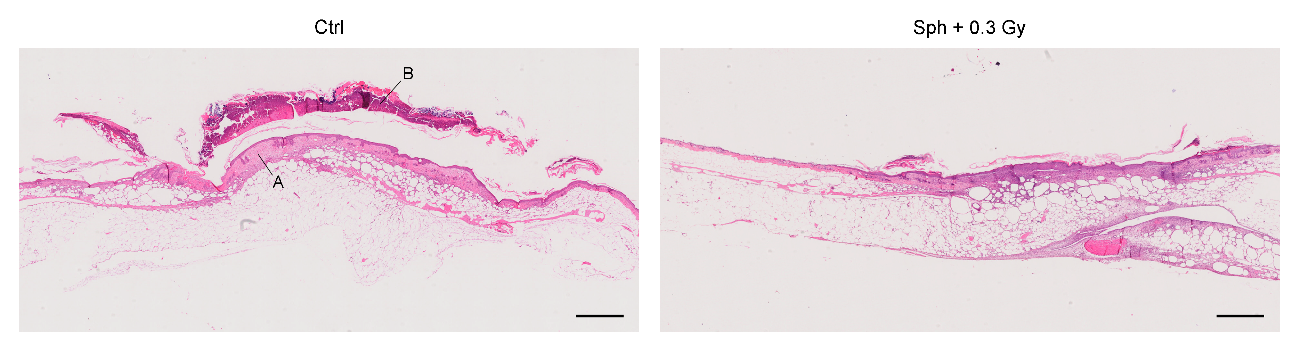


Supplementary Fig. 4. Representative histological features of skin regeneration in control (Ctrl) and HDF-ECFC spheroids combined with *in vivo* LDIR wounds (Sph + 0.3 Gy). For the diabetic wound model, regenerated skin tissue was collected 10 days postwounding for stereological histopathological analysis. Thickened dermis (A) and seropurulent crust (B) are observed exclusively in untreated control wounds. Images are representative of the findings quantified in Fig. 7. Scale bar: 1 mm.
